# Evaluation of the clinical validity of fixation accuracy using an open-face black mask in head stereotactic radiotherapy

**DOI:** 10.1007/s12194-026-01041-1

**Published:** 2026-03-25

**Authors:** Nanae Takahashi, Yutaka Naoi, Naoko Okano, Yuka Inage, Chie Kurokawa, Naoto Shikama

**Affiliations:** 1 Department of Radiology, Juntendo University Nerima Hospital, 3-1-10 Takanodai, Nerima- ku, Tokyo, 117-8521 Japan; 2 Department of Radiation Oncology, Juntendo University Graduate School of Medicine, 2-1- 1 Hongo, Bunkyo-ku, Tokyo, 113-8421 Japan; 3 Department of Radiological Technology, Faculty of Health Science, Juntendo University, 2- 1-1 Hongo, Bunkyo-ku, Tokyo, 113-8421 Japan

**Keywords:** surface-guided radiotherapy, open-face mask, stereotactic radiotherapy, intra-fractional motion

## Abstract

In stereotactic radiotherapy (SRT), precise alignment is crucial for treatment effectiveness and safety and has traditionally been achieved using cone beam computed tomography (CBCT). Recently, radiation-free optimal surface imaging (OSI) systems have gained popularity. Notably, the effectiveness of black face masks for identifying facial surfaces during OSI use has been increasingly reported. This study aimed to determine whether the fixation accuracy of an open-face black mask designed to facilitate detection using the OSI system is clinically acceptable. This study was approved by the institutional review board (protocol code E23-0162) on May 27th, 2023. We analyzed the accuracy and correlation of CBCT- and OSI-based measurements before and after treatment in 20 patients who underwent SRT using open-face black mask fixation. Additionally, intra-fractional motion was monitored in real time using the OSI system. Mean translational deviations measured by CBCT and OSI were 0.1 mm and 0.1 mm (lateral), -0.2 mm and 0.1 mm (longitudinal), and − 0.1 mm and 0.2 mm (vertical), respectively. Mean rotational deviations were 0.1° and − 0.1° (rotation), -0.4° and − 0.1° (roll), and 0.1° and − 0.1° (pitch). All measurements met clinical accuracy criteria. Weak correlations between CBCT and OSI data were observed only in the lateral and roll directions. Several transient outliers were noted in real-time OSI monitoring. The fixation accuracy achieved with the open-face black mask was within clinically acceptable limits based on both CBCT and OSI measurements. Further research is warranted to clarify the source of noise and improve the reliability of real-time OSI-based monitoring.

## Introduction

Stereotactic radiotherapy (SRT) is a standard treatment for brain tumors, including certain metastatic and benign tumors. This technique allows the delivery of high radiation doses with a dose distribution tailored to the shape of the tumor. In brain tumors, margins are often limited due to concerns about brain radiation necrosis and edema, making precise fixation and positioning critical [1–3].

Previously, head frames were used for precise head fixation, requiring an invasive procedure in which the frame was secured to the skull with screws [3, 4]. Although head frames offered superior accuracy, they were invasive and time-consuming to set up. Recent advances in positioning and irradiation techniques have increased the use of SRT with linear accelerators, which employ non-invasive immobilization systems such as thermoplastic masks [5, 6].

The optimal surface imaging (OSI) system uses a monitoring camera to detect the movement of the patient’s skin surface and calculate its misalignment. This system allows for monitoring the alignment during each treatment and identifies any misalignment during irradiation [7–10].

Phantom studies have shown that combining the ESS-50, an open-face black mask for head fixation, with the high-definition monitoring system Catalyst HD (C-RAD AB, Uppsala, Sweden) confirms fixation accuracy and reproducibility [11, 12]. Other studies have also explored the use of open-face masks in conjunction with the OSI system for head radiotherapy [13, 14].

This study aimed to verify the fixation accuracy of the ESS-50 open-face mask during irradiation in patients using the OSI system.

## Materials and methods

### Patients

From June 2023 to December 2024, patients receiving SRT for brain tumors were prospectively enrolled. Written informed consent for treatment was obtained from all patients. This study was approved by the institutional review board (IRB) of the authors (protocol code E23-0162).

### Devices and treatment details

**Fig. 1:**
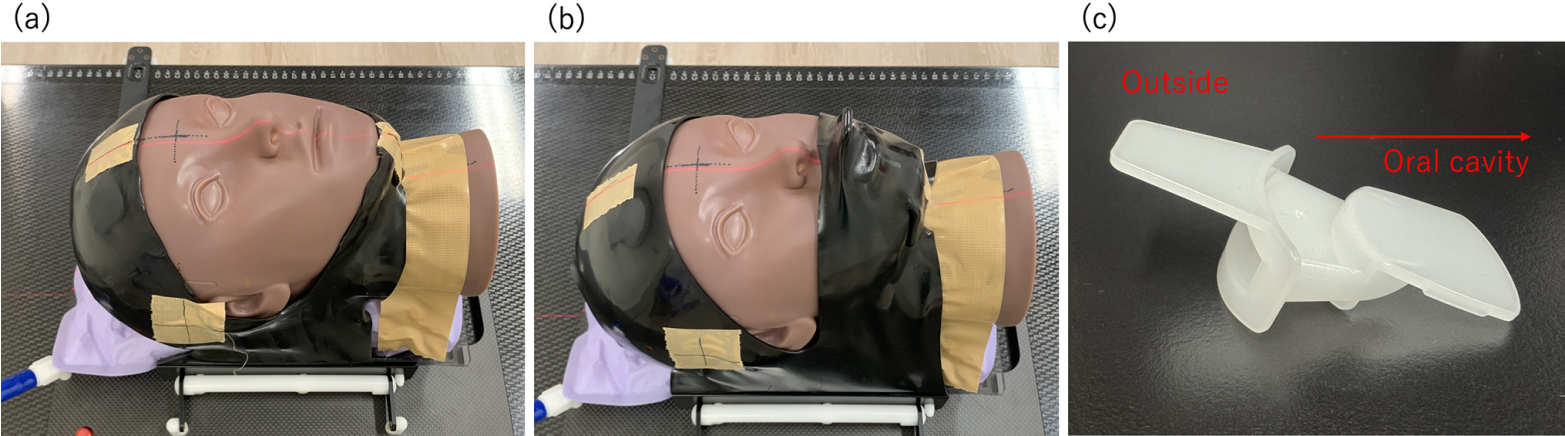
Images of immobilization system and open-face masks. (a) ESS-50: open type; (b) ESS-51: open type with mouthpiece; and (c) mouthpiece for ESS-51

The ESS-50, an open-face black mask for head fixation, and the ESS-51, an open-face black mask with a mouthpiece for head fixation, were used as head immobilization devices (Engineering Systems Inc.), along with a mouthpiece unless otherwise specified (Fig. 1). Treatment planning was performed using computed tomography (CT) with Aquilion LB (Canon Medical Systems Co. Tochigi, Japan) at 1 mm slice thickness. The treatment plan was created using Monaco software (Elekta AB, Stockholm, Sweden).

Contrast-enhanced T1-weighted magnetic resonance imaging at 1 mm thickness was fused to delineate the gross tumor volume (GTV). The clinical target volume (CTV) was defined as equal to the GTV, and the planning target volume (PTV) was created by adding a 2 mm margin around the CTV [15]. Dose fractionation was 32 Gy in 4 fractions for brain metastases and 20 Gy in 4 fractions for benign tumors. The prescribed dose was administered to 95% of the target volume. This study focuses on the treatment of single lesions, and SRT was performed using a single isocenter.

The treatment was delivered using a linear accelerator (Versa HD; Elekta AB, Stockholm, Sweden), with Cone Beam CT (XVI), and a HexaPOD evo couch (HPe), which allowed for 6-axis positional corrections. In the XVI system, positional inaccuracies of the kV-MV isocenter caused by gantry and imager deflection and gantry rotation direction are corrected using a function called Flex map, which represents positional deviation of the kV-MV isocenter as a function of gantry angle. This correction ensures that the positional accuracy of the kV-MV isocenter is within 0.5 mm of the acceptance test tolerance. The accuracy of the Flex map has been reported to be approximately 0.1–0.3 mm, with a maximum of 0.75 mm [16–18]. The OSI system used was Catalyst HD in high-definition mode. Catalyst HD is characterized by a measurement reproducibility of 0.2 mm and long-term stability of 0.3 mm. It operates at a frame rate of 200 frames per second, ensuring timely data collection. Additionally, the positional accuracy for rigid bodies is ≤ 0.5 mm, and the motion detection accuracy is also ≤ 0.5 mm, providing high precision and stability throughout the experimental procedures [19]. Cone beam computed tomography (CBCT) was performed before and after treatment to monitor intra-fractional motion, and the OSI system was used for continuous real-time motion monitoring during irradiation.

During irradiation, images from the monitoring camera were visually confirmed. If patient movement was observed, irradiation was temporarily halted, and a CBCT scan was performed to confirm that no displacement had occurred before resuming irradiation.

### Evaluation method

Intra-fractional motion was evaluated by measuring displacement values (deviations) before and after treatment using CBCT. Deviations were also measured using the OSI system. However, although the OSI system enables continuous monitoring, its termination timing does not coincide with CBCT acquisition, making it difficult to precisely identify the exact moment of CBCT imaging. Therefore, to reduce selection bias in the comparative analysis, the average deviation over the 5 s preceding CBCT acquisition was used. Deviations obtained from both methods were then compared.

### Statistical analysis

The Pearson correlation coefficient was used to compare deviations between CBCT and the OSI system. Statistical analysis was performed using R version 4.4.2 (R Core Team, Vienna, Austria) [20].

## Results

### Patient characteristics

Patient characteristics are shown in Table 1. Twenty patients were enrolled, with only one case treated using eight fractions. One case treated with eight fractions required an increased number of fractions due to the large tumor size and a recent history of stereotactic gamma knife irradiation near the treatment lesion. However, the treatment planning concept remained the same.

**Table 1:** Patient Characteristics and Treatment Details

		*N* = 20	
Age, years			
	Median, range	67	(38–79)
Diagnosis of brain tumor			
	Metastasis	17	85%
	Schwannoma	3	15%
Dose fractionation			
	32 Gy/ 4 fractions	14	70%
	20 Gy/ 4 fractions	5	25%
	32 Gy/ 8 fractions	1	5%
Use of non-coplanar beams			
	Yes	2	10%
	No	18	90%
Use of Mouse Piece			
	Yes	18	90%
	No	2	10%
Treatment time, sec			
	Median, range	215.1	(171–723)

Mouthpieces could not be used in two cases, and noncoplanar beams were used in two cases. One registered session was excluded from the analysis because data could not be acquired due to an interruption requested by the patient during irradiation.

### Displacement measurement of intra-fractional motion

The mean translational deviation measured by CBCT and the OSI system was 0.1 mm and 0.1 mm in the lateral direction, -0.2 mm and 0.1 mm in the longitudinal direction, and − 0.1 mm and 0.2 mm in the vertical direction, respectively. The mean rotational deviation measured by CBCT and the OSI system was 0.1° and − 0.1° in rotation, -0.4° and − 0.1° in roll, and 0.1° and − 0.1° in pitch, respectively (Table 2,Supplementary Table 1). CBCT measurements showed that differences before and after treatment met the criteria for stereotactic irradiation: translational deviation was within 0.5 mm in any direction, and rotational deviation was within 1° in all directions [21]. Measurements using the OSI system also met these criteria [21]. Comparison of CBCT and OSI data showed weak correlations in the lateral and roll directions, whereas no clear correlations were observed in the other directions.

**Table 2:** CBCT and the OSI system consistency check(*N* = 83 sessions)

		CBCT		OSI system		*R*-value	*P*-value
		Mean	SD	Mean	SD		
Translations, mm							< 0.01
	Lat.	0.1	0.5	0.1	0.2	0.415	
	Long.	-0.2	0.6	0.1	0.2	0.216	0.05
	Vert.	-0.1	0.3	0.1	0.2	0.267	0.01
Deviation, mm		0.6	0.6	0.3	0.2	0.024	0.83
Rotations: mean, degree							0.02
	Rot.	0.1	0.2	-0.1	0.2	-0.256	
	Roll	-0.4	0.3	-0.1	0.1	0.342	< 0.01
	Pitch	0.1	0.3	-0.1	0.2	-0.097	0.38

Abbreviations: CBCT, Cone Beam Computed Tomography; OSI system, Optical Surface System; Lat., Lateral; Long, Longitudinal; Vert., vertical; Rot., Rotation

### Real-time monitoring of intra-fractional motion

**Fig. 2:**
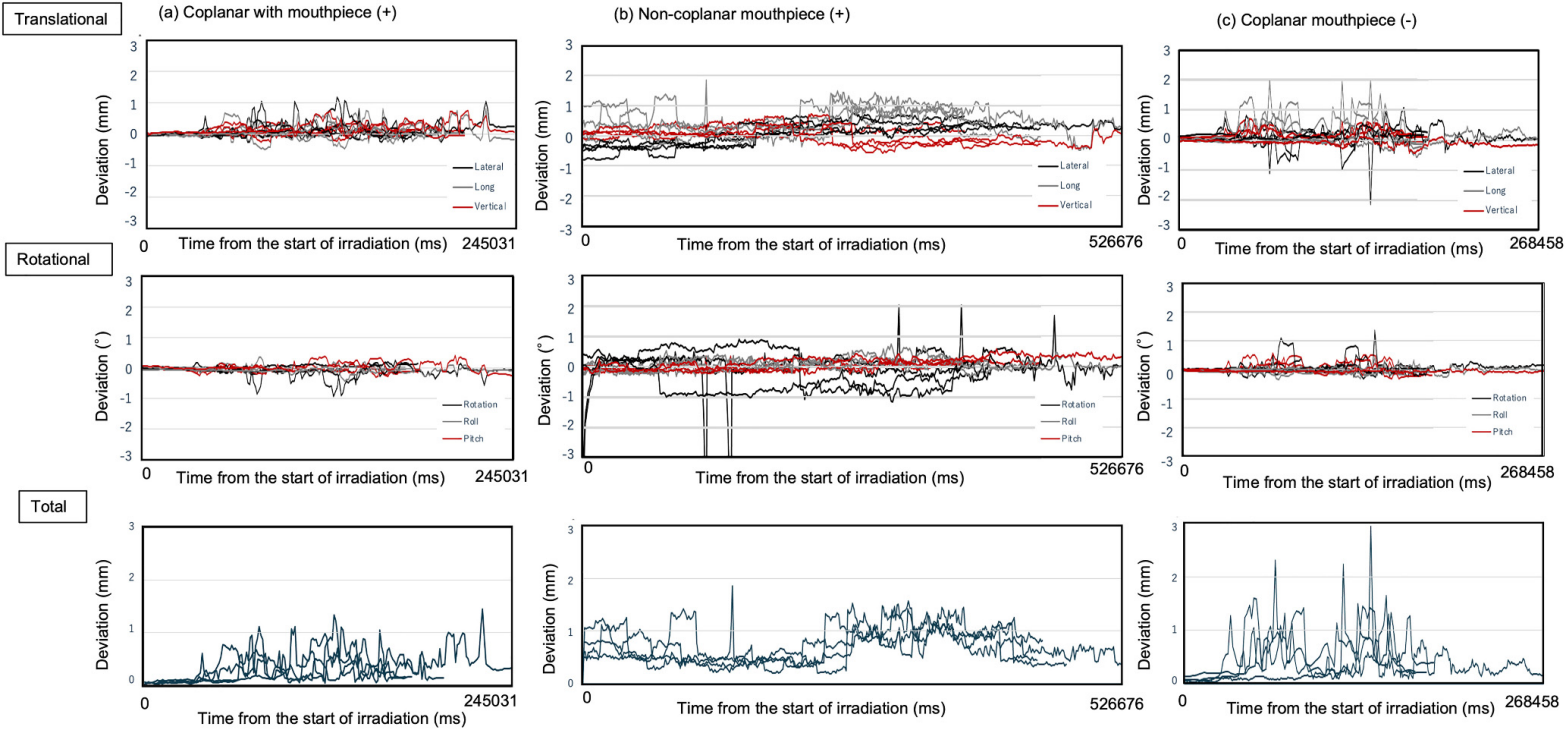
Trends of intra-fractional motion across all sessions for representative examples of each condition. Top: translational displacement, middle: rotational displacement, bottom: three-dimensional deviation. (a) Coplanar beam with ESS-51; (b) Noncoplanar beam with ESS-51; (c) coplanar beam with ESS-50

The absolute values of the mean and maximum translational deviation were 0.1 mm and 2.2 mm in the lateral direction, 0.1 mm and 2.6 mm in the longitudinal direction, and 0.1 mm and 1.4 mm in the vertical direction, respectively. The absolute values of mean and maximum rotational deviation were 0.1° and 1.9° in rotation, 0.0° and 1.3° in roll, and 0.1° and 1.5° in pitch, respectively (Table 3,Supplementary Table 2). During visual monitoring of irradiation, no patient movement was observed except in one case where irradiation was interrupted at the patient’s request. Real-time monitoring logs of representative cases are shown in Fig. 2. They demonstrate that significant deviations were temporary and did not persist throughout treatment.

**Table 3:** The intra-fractional motion Monitoring by OSI system. (*N* = 83 sessions)

	Translation, mm		Vert.		Rotation, degree		Pitch		3D Deviation, mm	
	Lat.	Long.			Rot.	Roll				
Mean, ABS	0.1	0.1	0.1		0.1	0.0	0.1		0.2	
SD	0.2	0.4	0.1		0.2	0.1	0.2		0.5	
Maximum, ABS	2.2	2.6	1.4		1.9	1.3	1.5		3.5	
SD	6.6	8.6	3.9		6.7	4.8	5.7		10.4	

SD, Standard Deviation; ABS, Absolute Value; Lat., Lateral; Long, Longitudinal; Vert., Vertical; Rot., Rotation; 3D, 3-Dimentional

## Discussion

In this study, clinical cases of cranial SRT were treated using open-face black masks (ESS-50 and ESS-51), and intra-fractional motion was measured using both CBCT and the OSI system (Catalyst HD). The average deviation remained within the American Association of Physicists in Medicine (AAPM) TG-198 guidelines for stereotactic radiosurgery (SRS) [22].

Babic et al. evaluated intra-fractional motion using CBCT across different immobilization methods, reporting mean deviations of 0.30–0.54 mm with a frame and 0.73–0.76 mm with a plastic mask [6]. Chen et al. reported a mean bias of intra-fractional motion using an open-face mask and the OSI system with non-coplanar beams of 0.01 cm, -0.07 cm, 0.03 cm, -0.30°, -0.08° and 0.00°, respectively [13]. Sanchez-Rubio et al. reported a mean intra-fractional motion of 3.04 mm with an open-face mask and surface-guided radiation therapy (SGRT) [14]. In the present study, the mean intra-fractional motion was 0.6 mm with CBCT and 0.2 mm with SGRT, showing accuracy comparable to previous reports. These results suggest that the open-face black masks (ESS-50 and ESS-51) provide excellent fixation performance comparable to other immobilization devices. Regarding mask color, this study used black masks, whereas other reports have employed masks of different colors. Kojima et al. reported that black masks are preferable when combined with Catalyst HD because they enable more accurate recognition of the patient’s body surface compared with white masks [11]. Since this study used only black masks, it is difficult to evaluate advantages based on color tone; however, mask color may have contributed to the comparable accuracy achieved. Compared to the phantom studies by Kojima et al. and Inage et al., which used the same system, deviations in the present clinical study were overall larger [11, 12]. Nevertheless, they remained within the limits recommended by the AAPM TG-198 report for SRS [22].

Regarding the consistency between CBCT and OSI system results, several previous reports have highlighted differences in displacement measurements between CBCT and SGRT [14, 21, 23–27]. These include OSI systems such as AlignRT (VisionRT Ltd, London, UK) and Catalyst HD. In general, CBCT tends to detect larger deviations, and minimal correlation has been observed between measurements from the two systems. This trend was also seen in the present study and is believed to reflect the inherent differences in measurement characteristics between CBCT and SGRT.

In real-time monitoring data, mean deviations remained within the tolerance range; however, maximum deviations occasionally exceeded 1 mm. In all such cases, deviations exceeding 1 mm were transient. We evaluated the impact of these transient deviations on dose distribution. This study employed volumetric modulated arc therapy (VMAT), in which the monitor units (MU) per gantry angle in the average plan accounted for approximately 0.2% of the total MU. Since angular displacement during momentary shifts rarely exceeds a few degrees, the overall dose deviation remains below 1%, which is considered clinically acceptable. On the other hand, it has been suggested that such transient deviations may represent noise from the OSI system. This phenomenon was not observed in phantom studies, indicating that it may be specific to the clinical setting [11, 12]. We suspect gantry interference as a possible cause. Since this study employed rotational irradiation, interference between the gantry and the camera may have caused temporary reduction in data volume, resulting in measurement errors. Further investigation is needed to confirm this hypothesis. Most cases in this study were treated using coplanar beams alone with the patient wearing a mouthpiece. Noncoplanar beams were used in two cases, and mouthpieces were not used in two cases. Owing to the small number of cases in each group, statistical evaluation was difficult. However, as shown in Fig. 2, deviations tended to be larger in cases treated with noncoplanar beams or without a mouthpiece. Furthermore, as treatment time increased, a tendency toward greater deviation was observed, although this advantage was not significant in the latter half of treatment. With respect to time-related deviation, the influence of thermal drift in the OSI system camera has been noted [28]. Therefore it was difficult to determine whether the observed deviation originated from patient movement or the OSI system itself.

A limitation of this study is that no clinically significant displacements were observed in the current case series, making it difficult to evaluate the ability of SGRT to detect large displacements.　Additionally, because the evaluation focused on values close to the accuracy limits of CBCT and the OSI system, it remains unclear whether the observed discrepancies were due to system errors or limitations in measurement precision. Nonetheless, all results were within clinically acceptable ranges, and the accuracy of the fixation device used in this study remains valid.

## Conclusion

In clinical settings, the accuracy of the open-face masks ESS-50 and ESS-51 was considered acceptable based on measurements from both CBCT and the OSI system. However, no strong correlation was observed between the results obtained from the two systems. Regarding the accuracy of real-time intra-fractional motion using the OSI system, further investigation is needed to identify sources of noise and develop methods for their elimination.

## Data Availability

The data supporting the findings of this study are not publicly available; however, they are available from the corresponding author upon reasonable request.

## References

[1] Schell MC, Bova FJ, Larson DA, Leavitt DD, Lutz WR, Podgorsak EB, et al. Stereotactic radiosurgery. American Association of Physicists in Medicine Report No. 54. Woodbury, NY: American Institute of Physics; 1995. pp. 1–88.

[2] Kirkpatrick JP, Wang Z, Sampson JH, McSherry F, Herndon JE, Allen KJ, et al. Defining the optimal planning target volume in image-guided stereotactic radiosurgery of brain metastases: results of a randomized trial. Int J Radiat Oncol Biol Phys. 2015;91:100–8. 10.1016/j.ijrobp.2014.09.004. 25442342 10.1016/j.ijrobp.2014.09.004

[3] Lightstone AW, Benedict SH, Bova FJ, Solberg TD, Stern RL. Intracranial stereotactic positioning systems: report of the American Association of Physicists in Medicine Radiation Therapy Committee Task Group No. 68. Med Phys. 2005;32:2380–98. 10.1118/1.1945347, PMID: 28493584. 10.1118/1.1945347 28493584

[4] Ertl A, Saringer W, Heimberger K, Kindl P. Quality assurance for the Leksell gamma unit: considering magnetic resonance image-distortion and delineation failure in the targeting of the internal auditory canal. Med Phys. 1999;26:166–70. 10.1118/1.598499, PMID: 10076969. 10.1118/1.598499 10076969

[5] Wen N, Li H, Song K, Chin-Snyder K, Qin Y, Kim J, et al. Characteristics of a novel treatment system for linear accelerator-based stereotactic radiosurgery. J Appl Clin Med Phys. 2015;16:125–48. 10.1120/jacmp.v16i4.5313. PMID: 26218998, PMCID: PMC5690003. 26218998 10.1120/jacmp.v16i4.5313 PMC5690003

[6] Babic S, Lee Y, Ruschin M, Lochray F, Lightstone A, Atenafu E, et al. To frame or not to frame? Cone-beam CT-based analysis of head immobilization devices specific to linac-based stereotactic radiosurgery and radiotherapy. J Appl Clin Med Phys. 2018;19:111–20. 10.1002/acm2.12251. [EPub]. PMID: 29363282, PMCID: PMC5849846. 29363282 10.1002/acm2.12251 PMC5849846

[7] Hoisak JDP, Pawlicki T. The role of optical surface imaging systems in radiation therapy. Semin Radiat Oncol. 2018;28:185–93. 10.1016/j.semradonc.2018.02.003. 29933878 10.1016/j.semradonc.2018.02.003

[8] Stieler F, Wenz F, Shi M, Lohr F. A novel surface imaging system for patient positioning and surveillance during radiotherapy. A phantom study and clinical evaluation. Strahlenther Onkol. 2013;189:938–44. 10.1007/s00066-013-0441-z. 24068172 10.1007/s00066-013-0441-z

[9] Walter F, Freislederer P, Belka C, Heinz C, Söhn M, Roeder F. Evaluation of daily patient positioning for radiotherapy with a commercial 3D surface-imaging system (Catalyst™). Radiat Oncol. 2016;11:154. 10.1186/s13014-016-0728-1. 27881158 10.1186/s13014-016-0728-1 PMC5122202

[10] Carl G, Reitz D, Schönecker S, Pazos M, Freislederer P, Reiner M, et al. Optical surface scanning for patient positioning in radiation therapy: A prospective analysis of 1902 fractions. Technol Cancer Res Treat. 2018;17:1533033818806002. 10.1177/1533033818806002. 30453842 10.1177/1533033818806002 PMC6243634

[11] Kojima H, Takemura A, Kurokawa S, Ueda S, Noto K, Yokoyama H, et al. Evaluation of technical performance of optical surface imaging system using conventional and novel stereotactic radiosurgery algorithms. J Appl Clin Med Phys. 2021;22:58–68. 10.1002/acm2.13152. [EPub] 2020 Dec 27. PMID: 33369014, PMCID: PMC7882109. 33369014 10.1002/acm2.13152 PMC7882109

[12] Inage Y, Kurokawa C, Doryo K, Naoi Y. A study on LINAC couch position for brain stereotactic radiotherapy using high-definition optical surface imaging system. Nihon Hoshasen Gijutsu Gakkai Zasshi. 2025;81. 10.6009/jjrt.25-1540. Japanese. PMID: 40571601. 10.6009/jjrt.25-1540 40571601

[13] Chen X, Liu L, Wang Y, Huang X, Cai W, Rong X et al. Surface guided radiation therapy with an innovative open-face mask and mouth bite: patient motion management in brain stereotactic radiotherapy. Clin Transl Oncol. 2024;26:424–33. 10.1007/s12094-023-03260-z [EPub] 2023 Jul 3. PMID: 37395988. 10.1007/s12094-023-03260-z 37395988

[14] Sánchez-Rubio P, Rodríguez-Romero R, Pinto-Monedero M, Alejo-Luque L, Martínez-Ortega J. New findings on clinical experience on surface-guided radiotherapy for frameless non-coplanar stereotactic radiosurgery treatments. J Appl Clin Med Phys. 2024;25:e14510. 10.1002/acm2.14510. [EPub]. PMID: 39287562, PMCID: PMC11633809. 39287562 10.1002/acm2.14510 PMC11633809

[15] Naoi Y, Kunishima N, Yamamoto K, Yoda K. A planning target volume margin formula for hypofractionated intracranial stereotactic radiotherapy under cone beam CT image guidance with a six-degrees-of-freedom robotic couch and a mouthpiece-assisted mask system: a preliminary study. Br J Radiol. 2014;87:20140240. 10.1259/bjr.20140240. 25029296 10.1259/bjr.20140240 PMC4453155

[16] Chojnowski JM, Warr GB, Sykes JR, Thwaites DI. Assessment of error in the MV radiation isocenter position calculated with the Elekta XVI software. J Appl Clin Med Phys. 2020;21:93–7. 10.1002/acm2.12861. 32239750 10.1002/acm2.12861 PMC7286015

[17] Riis HL, Moltke LN, Zimmermann SJ, Ebert MA, Rowshanfarzad P. Investigation of the accuracy of MV radiation isocentre calculations in the Elekta cone-beam CT software XVI. Phys Med Biol. 2016;61:N249–56. 10.1088/0031-9155/61/11/N249. 27183466 10.1088/0031-9155/61/11/N249

[18] Yamazawa Y, Osaka A, Fujii Y, Nakayama T, Nishioka K, Tanabe Y. Evaluation of the effect of sagging correction calibration errors in radiotherapy software on image matching. Phys Eng Sci Med. 2024;47:589–96. 10.1007/s13246-024-01388-y. 38372942 10.1007/s13246-024-01388-y PMC11166816

[19] Hoisak JDP, Paxton AB, Waghorn B, Pawlicki T. Surface guided radiation therapy. CRC; 2021.

[20] R Core Team. R: A Language and Environment for Statistical Computing. Vienna, Austria: R Foundation for Statistical Computing; 2024. https://www.R-project.org/.

[21] Bry V, Saenz D, Pappas E, Kalaitzakis G, Papanikolaou N, Rasmussen K. End to end comparison of surface-guided imaging versus stereoscopic X-rays for the SRS treatment of multiple metastases with a single isocenter using 3D anthropomorphic gel phantoms. J Appl Clin Med Phys. 2022;23:e13576. 10.1002/acm2.13576. 35322526 10.1002/acm2.13576 PMC9121024

[22] Hanley J, Dresser S, Simon W, Flynn R, Klein EE, Letourneau D, et al. AAPM Task Group 198 Report: an implementation guide for TG 142 quality assurance of medical accelerators. Med Phys. 2021;48:e830–85. 10.1002/mp.14992. 34036590 10.1002/mp.14992

[23] Lai JL, Liu SP, Jiang XX, Liu J, Li A, Li B, et al. Can optical surface imaging Replace non-coplanar cone-beam computed tomography for non-coplanar set-up verification in single-isocentre non-coplanar stereotactic radiosurgery and hypofractionated stereotactic radiotherapy for single and multiple brain metastases? *Clin Oncol (R Coll Radiol)*. Clin Oncol (R Coll Radiol). 2023;35:e657–65. 10.1016/j.clon.2023.09.007. 37778972 10.1016/j.clon.2023.09.007

[24] Tonkin K, Goodall SK. Accuracy of the catalyst surface guidance system for patient monitoring during cranial SRS treatments. Phys Eng Sci Med. 2023;46:633–43. 10.1007/s13246-023-01238-3. [EPub]. PMID: 36971948. 36971948 10.1007/s13246-023-01238-3

[25] Covington EL, Fiveash JB, Wu X, Brezovich I, Willey CD, Riley K, et al. Optical surface guidance for submillimeter monitoring of patient position during frameless stereotactic radiotherapy. J Appl Clin Med Phys. 2019;20:91–8. 10.1002/acm2.12611. 31095866 10.1002/acm2.12611 PMC6560239

[26] Zhou S, Li J, Zhu X, Du Y, Yu S, Wang M, et al. Initial clinical experience of surface guided stereotactic radiation therapy with open-face mask immobilization for improving setup accuracy: a retrospective study. Radiat Oncol. 2022;17:104. 10.1186/s13014-022-02077-4. 35659685 10.1186/s13014-022-02077-4 PMC9167505

[27] Lee SK, Huang S, Zhang L, Ballangrud AM, Aristophanous M, Cervino Arriba LI, et al. Accuracy of surface-guided patient setup for conventional radiotherapy of brain and nasopharynx cancer. J Appl Clin Med Phys. 2021;22:48–57. 10.1002/acm2.13241. 33792186 10.1002/acm2.13241 PMC8130230

[28] Lehmann J, Standen TS, Kaur G, Wolf J, Wilfert A, Simpson J. Methodology of thermal drift measurements for surface guided radiation therapy systems and clinical impact assessment illustrated on the C-Rad Catalyst^+^ HD system. Tech Innov Patient Support Radiat Oncol. 2022;21:58–63. 10.1016/j.tipsro.2022.02.005. 35243046 10.1016/j.tipsro.2022.02.005 PMC8885575

